# Supplementary material for: Associations of psychosocial factors and cardiovascular health measured by Life’s Essential 8: The Atherosclerosis Risk in Communities (ARIC) study
Source: PLoS One. 2024 Jul 31;19(7):e0305709. doi: 10.1371/journal.pone.0305709 (PMC11290690; doi:10.1371/journal.pone.0305709)
Supplement: S1 Table — 2022 [7] and the scoring used in the Atherosclerosis Risk in Communities (ARIC) study based on diet item availability. (DOCX) [file pone.0305709.s001.docx]

| **S1 Table.** Details of diet scoring, comparing the foods included on the Life’s Essential 8 scoring by Lloyd-Jones et al. 2022 and the scoring used in the Atherosclerosis Risk in Communities (ARIC) Study based on diet item availability. | | | |
| --- | --- | --- | --- |
| **Diet Component** | **Life’s Essential 8 Foods** | **ARIC Foods** | **Scoring** |
| Fruits | Fruits and fruit juices | Apples/pears, oranges, oranges/grapefruit juice, peaches/apricots/plums, bananas, other fruits | 1 = Quintile 1  2 = Quintile 2  3 = Quintile 3  4 = Quintile 4  5 = Quintile 5 |
| Vegetables | Vegetables (except potatoes and legumes) | Green beans, broccoli, cabbage/cauliflower/brussels sprouts, carrots, corn, spinach/collards, squash, sweet potatoes, tomatoes |  |
| Nuts and legumes | Nuts, peanut butter, dried beans, peas, tofu | Peas/lima beans, beans/lentils, peanut butter, nuts |  |
| Whole grains | Brown rice, dark breads, cooked cereal, whole grain cereal, other grains, popcorn, wheat germ, bran | Cooked cereal, dark/whole grain bread |  |
| Low-fat dairy | Skim milk, yogurt, cottage cheese | Skim milk, yogurt, cottage cheese |  |
| Sodium | Sum of all sodium content | Sum of all sodium content | 1 = Quintile 5  2 = Quintile 4  3 = Quintile 3  4 = Quintile 2  5 = Quintile 1 |
| Red and processed meats | Beef, pork, lamb, deli meats, organ meats, hot dogs, bacon | Hamburgers, hot dogs, processed meats, bacon, beef/pork/lamb sandwich, beef/pork/lamb dish |  |
| Sweetened beverages | Carbonated and non-carbonated sweetened beverages | Low calorie soft drinks, regular soft drinks, fruit-flavored punch/non-carbonated beverages |  |
| Total diet score calculated by summing together scores for all 8 diet components. | | | |
