# Supplementary material for: Associations of psychosocial factors and cardiovascular health measured by Life’s Essential 8: The Atherosclerosis Risk in Communities (ARIC) study
Source: PLoS One. 2024 Jul 31;19(7):e0305709. doi: 10.1371/journal.pone.0305709 (PMC11290690; doi:10.1371/journal.pone.0305709)
Supplement: S2 Table — (DOCX) [file pone.0305709.s002.docx]

| **S2 Table**. Cross-sectional adjusted associations of psychosocial factors with cardiovascular health defined by the American Heart Association’s Life’s Essential 8 metric at ARIC Visit 2, using multivariable linear and logistic regressions; N=11,311 | | |
| --- | --- | --- |
|  | **N** | **β (95% CI)** |
| **Social isolation** |  |  |
| Isolated/high (0-25) | 599 | -2.16 (-3.30, -1.03) |
| Moderate (26-30) | 1471 | -1.07 (-1.83, -0.32) |
| Low (31-50) | 9241 | Referent |
| **Social support** |  |  |
| High (41-48) | 4062 | Referent |
| Moderate (36-40) | 3357 | -1.62 (-2.25, -1.00) |
| Low (4-35) | 3892 | -4.27 (-4.87, -3.66) |
| **Trait anger** |  |  |
| High (22-40) | 787 | -6.09 (-7.12, -5.05) |
| Moderate (15-21) | 6268 | -2.85 (-3.38, -2.32) |
| Low (10-14) | 4256 | Referent |
| **Depressive symptoms** |  |  |
| High (10-36) | 3865 | -8.22 (-8.85, -7.58) |
| Moderate (4-9) | 3815 | -3.16 (-3.77, -2.55) |
| Low (0-3) | 3631 | Referent |
| ARIC: Atherosclerosis Risk in Communities Study; β: beta; CI: confidence interval  Models adjusted for sex, race-center, age, and education. | | |
