# Supplementary material for: Associations of psychosocial factors and cardiovascular health measured by Life’s Essential 8: The Atherosclerosis Risk in Communities (ARIC) study
Source: PLoS One. 2024 Jul 31;19(7):e0305709. doi: 10.1371/journal.pone.0305709 (PMC11290690; doi:10.1371/journal.pone.0305709)
Supplement: S3 Table — (DOCX) [file pone.0305709.s003.docx]

| **S3 Table**. Cross-sectional adjusted associations of psychosocial factors with cardiovascular health, defined by Life’s Essential 8, and Life’s Essential 8 sleep score, comparing scores defined using hours of sleep and sleep quality; N=1,578 | | | | |
| --- | --- | --- | --- | --- |
|  | Hours of sleep measures | | Sleep quality measures | |
|  | CVH score | High CVH sleep | CVH score | High CVH sleep |
|  | β (95% CI) | β (95% CI) | β (95% CI) | β (95% CI) |
| **Social isolation** |  |  |  |  |
| Isolated/high (n=61) | -1.49 (-4.84, 1.87) | -10.77 (-17.12, -4.42) | -0.84 (-4.30, 2.61) | -5.64 (-13.15, 1.86) |
| Moderate (n=165) | -0.40 (-2.52, 1.72) | 0.53 (-3.49, 4.54) | -1.10 (-3.28, 1.08) | -5.08 (-9.83, -0.34) |
| Low (n=1352) | Referent | Referent | Referent | Referent |
| **Social support** |  |  |  |  |
| High (n=583) | Referent | Referent | Referent | Referent |
| Moderate (n=473) | -0.97 (-2.66, 0.62) | -1.24 (-4.26, 1.78) | -1.61 (-3.23, 0.02) | -6.35 (-9.86, -2.83) |
| Low (522) | -2.27 (-3.82, -0.72) | -1.51 (-4.46, 1.44) | -3.49 (-5.08, -1.90) | -11.25 (-14.68, -7.81) |
| **Trait anger** |  |  |  |  |
| High (n=86) | -2.95 (-5.93, 0.03) | 0.86 (-4.79, 6.52) | -4.90 (-7.94, -1.85) | -14.72 (-21.33, -8.11) |
| Moderate (n=935) | -1.76 (-3.14, -0.38) | -0.09 (-2.71, 2.53) | -2.61 (-4.02, -1.20) | -6.87 (-9.93, -3.81) |
| Low (n=557) | Referent | Referent | Referent | Referent |
| **Depressive symptoms** |  |  |  |  |
| High (n=432) | -3.68 (-5.36, -2.01) | -3.44 (-6.63, -0.26) | -7.07 (-8.76, -5.37) | -30.51 (-33.96, -27.06) |
| Moderate (n=561) | -2.04 (-3.56, -0.51) | -1.28 (-4.18, 1.61) | -3.52 (-5.06, -1.98) | -13.12 (-16.26, -9.98) |
| Low (n=585) | Referent | Referent | Referent | Referent |
| β: beta; CI: confidence interval  Models adjusted for sex, race-center, age, and education.  High cardiovascular health: Life’s Essential 8 score 80-100  Social isolation: low risk ≥ 31, 25 < moderate risk ≤ 30, high risk/socially isolated ≤ 25  Social support: 4 ≤ low < 36, 36 ≤ moderate < 41, 41 ≤ high ≤ 48  Trait anger: 10 ≤ low < 15, 15 ≤ moderate < 22, 22 ≤ high ≤ 40  Depressive symptoms: 0 ≤ low < 4, 4 ≤ moderate < 10, 10 ≤ high ≤36 | | | | |
